# Variability of plant transcriptomic responses under stress acclimation: a review from high throughput studies

**DOI:** 10.3389/abp.2024.13585

**Published:** 2024-10-25

**Authors:** Michał Rurek, Mikołaj Smolibowski

**Affiliations:** Department of Molecular and Cellular Biology, Institute of Molecular Biology and Biotechnology, Faculty of Biology, Adam Mickiewicz University, Poznań, Poland

**Keywords:** acclimation, differentially expressed genes, plant transcriptome, RNA-seq, stress response

## Abstract

Plant transcriptomes are complex entities shaped spatially and temporally by a multitude of stressors. The aim of this review was to summarize the most relevant transcriptomic responses to selected abiotic (UV radiation, chemical compounds, drought, suboptimal temperature) and biotic (bacteria, fungi, viruses, viroids) stress conditions in a variety of plant species, including model species, crops, and medicinal plants. Selected basic and applicative studies employing RNA-seq from various sequencing platforms and single-cell RNA-seq were involved. The transcriptomic responsiveness of various plant species and the diversity of affected gene families were discussed. Under stress acclimation, plant transcriptomes respond particularly dynamically. Stress response involved both distinct, but also similar gene families, depending on the species, tissue, and the quality and dosage of the stressor. We also noted the over-representation of transcriptomic data for some plant organs. Studies on plant transcriptomes allow for a better understanding of response strategies to environmental conditions. Functional analyses reveal the multitude of stress-affected genes as well as acclimatory mechanisms and suggest metabolome diversity, particularly among medicinal species. Extensive characterization of transcriptomic responses to stress would result in the development of new cultivars that would cope with stress more efficiently. These actions would include modern methodological tools, including advanced genetic engineering, as well as gene editing, especially for the expression of selected stress proteins *in planta* and for metabolic modifications that allow more efficient synthesis of secondary metabolites.

## Introduction

Higher plants, known as vascular or telome plants (Thelomophyta), appeared during plant evolution back in the Palaeophytic era. They are characterized by the development of tissues that distribute water, mineral compounds, and photosynthesis products, and the dominance of the sporophyte (Kenrick and Crane, 1997; Forster et al., 2007). Due to the sessile life cycle, higher plants respond adequately to unfavorable conditions at multiple levels, including transcriptomic one (Morris et al., 2018).

The plant transcriptome is a complete pool of various RNA molecules (mRNA, rRNA, tRNA, as well as numerous ncRNAs) belonging to the translated fraction of the genome that responds to its environment (Imadi et al., 2015). Transcriptomics belongs to key “omics” studies that link genomic and proteomic “worlds” by analyses of RNA, a biopolymer with a central role in the transfer of genetic information and regulation of gene expression. Transcriptomics shows a more universal status than other “omics” disciplines. It offers complex and deep insights into studying gene expression in whole plants or plant organs/tissues; factors that regulate the transcriptome spatially and temporally can also be characterized (Alkan et al., 2011; Lowe et al., 2017; Zhang, 2019; Athanasopoulou et al., 2021). Moreover, plant genome assembly is more complex and expensive compared to RNA sequencing (RNA-seq), and when a reference genome is absent, the transcriptome can be used to assess plant overall transcriptional activity. Transcriptomics also allows for the quantification of low-abundance transcripts or their structural variants and estimation of the correlation of gene expression with biological traits. Additionally, the transcriptome outperforms the genome, allowing the characterization of genes related to therapeutic compound biogenesis (Wang et al., 2009). However, transcriptomics seems to be inappropriate for identifying genes with large impacts on adaptive responses to the environment due to a small number of genes with large impacts on fitness. When only transcriptomics is used to identify genes underlying environmental adaptations, constitutively expressed regulatory genes that play a major role in setting tolerance limits are often over-represented (Evans, 2015). Other disadvantages are serious challenges in analysing large datasets, as they demand a lot of bioinformatic tools, and most importantly, costs of sequencing (discussed below). The transcriptomic data may also contain noise enhanced by technical variations and batch effects resulting from inter-sample differences that were not rooted in the experimental design (Sprang et al., 2022).

Tissue-specific transcriptomics offer particularly valuable information on the underlying molecular processes that govern tissue-specific functions; furthermore, specific genes and regulatory mechanisms that display unique roles in diverse tissues can be better characterized (Booth et al., 2022). Droplet single cell RNA-seq (with most prominent platforms, including 10x Genomics) and spatial RNA-seq (with microdissection, spatial imaging and spatial coding approaches) allow the possibility of getting insight into the heterogeneity of tissue transcriptomes, to identify cell types and markers, and to analyse gene and regulatory networks under developmental and environmental factors. Their strengths include, for instance, the availability of spatial information and high resolution performance (Cervantes-Pérez et al., 2022; Chen et al., 2023; Wang et al., 2024).

The recent and prompt development of high-throughput RNA-seq platforms with a subsequent decrease in sequencing costs, as well as data meta-analyses, advanced on plant transcriptome studies (Tyagi et al., 2022). Currently, third-generation sequencing, including SMRT (single-molecule real-time) and Nanopore sequencing allows to obtain longer sequence reads, which challenged transcriptomic analyses. Long read sequencing is accurate and allows detection of alternative splicing events. SMRT sequencing employs sequencing by synthesis, linking of chemical groups to reduce background noise and is based on properties of zero-mode waveguides. In SMRT protocol, there is no need for amplification. SMRT sequencing was used by Pacific Biosciences of California (PacBio) platform. Nanopore platform is based on electrical signal sequencing and offers particularly long reads (Li et al., 2018; Ma L. N. et al., 2019; Huang et al., 2021). However, Illumina RNA-seq is still the most preferred sequencing platform in quantitative analyses (Supplementary Table S1).

The study of tissue-specific changes in gene expression under stress is instrumental in the development of strategies to improve plant response under environmental conditions (Berkowitz et al., 2021; Tyagi et al., 2022). In previous years, some reviews focused on methodological advances in plant transcriptomics (Schliesky et al., 2012; Tyagi et al., 2022; Chen et al., 2023; Wang et al., 2024). Species-specific omics analyses characterized the relevance of transcription factors (TFs), hormones, translational reprogramming and epigenetic level, as well as phenotypic and physiological levels in stress response (Singh et al., 2016; Ahmad, 2022; Bhat et al., 2022; Hu et al., 2022; Kourani et al., 2022; Son and Park, 2023; Tu et al., 2023). Some studies also focused on the roles of non-coding RNA in stress response (Yu et al., 2019; Jin et al., 2024). However, an updated review discussing transcriptomic responses to various stress conditions assayed by high-throughput approaches from various plant species is currently needed.

**FIGURE 1:**
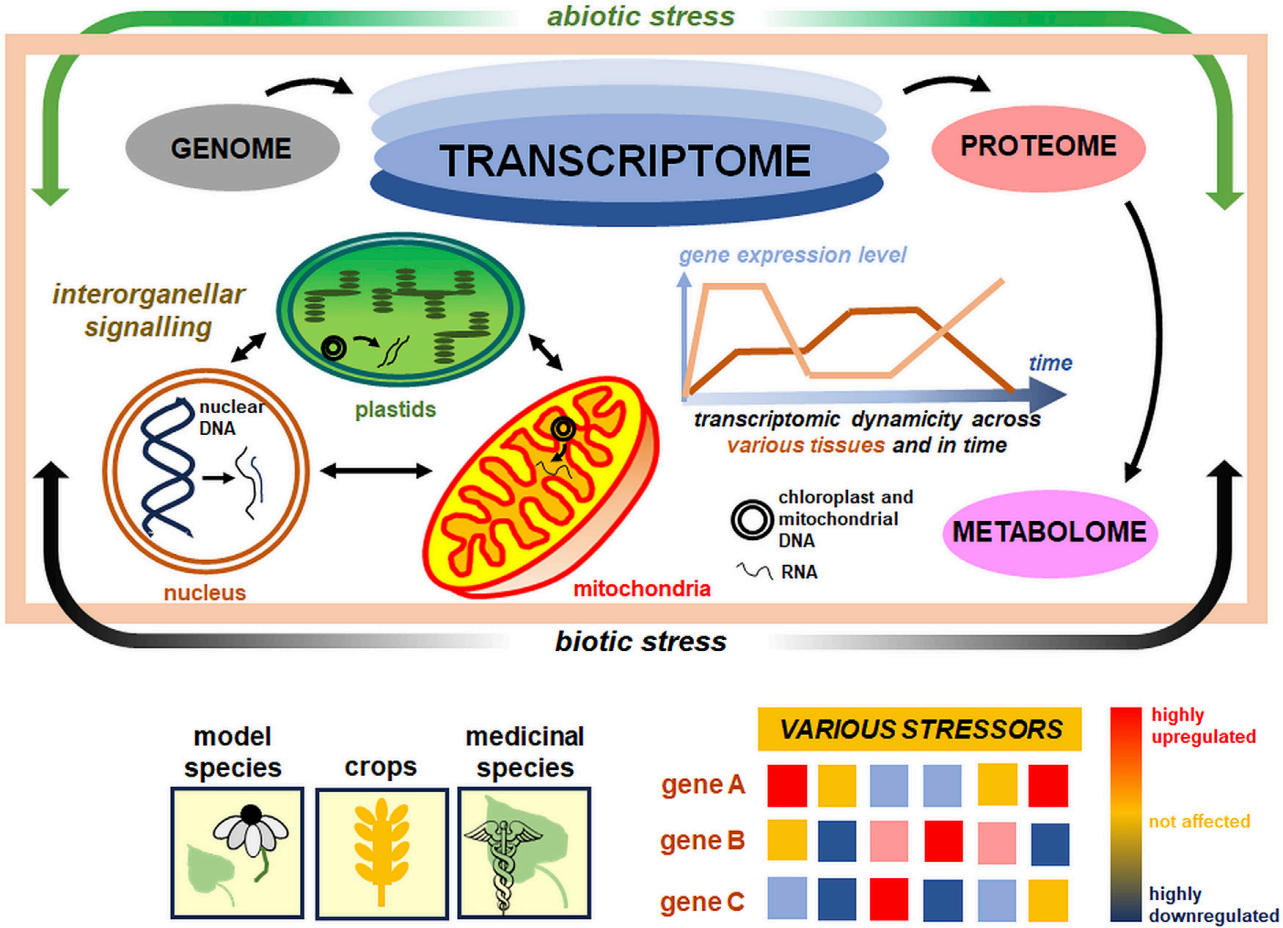
Plant transcriptome as a central stress-responsive entity in the cell. The plant transcriptome is a highly dynamic structure in plant cells (*bluish ovals at the top*). It links genomic, proteomic, and metabolomic levels, responding on a multidimensional scale, across various tissues, and along time (*the center*). The response of plant nuclear and organellar transcriptomes is also shaped by a number of factors, including abiotic and biotic stressors (*green and black arrows encompassing the light brown central rectangle*), which affect the differential expression pattern of various gene sets (*bottom*). For proper organellar biogenesis under stress acclimation, inter-organellar signaling between actively transcribed nuclear, plastid, and mitochondrial genomes is indispensable (*small arrows within marked organelles on the panel in the center and to the left*). The diversity of transcriptomes from model, crop, and medicinal species (*bottom*) under selected stress conditions was discussed in this paper. More details in the text.

In this paper, the diversity of transcriptomic responses to various stressors, both abiotic (including chemical treatments, UV radiation, drought, cold and heat) and biotic (fungal, bacterial, and viral/viroid infections) ones, will be presented, and the results will be discussed in tissue/developmental and temporal contexts, reflecting the transcriptomic dynamicity. We will focus on current studies employing high-throughput analyses, for instance, RNA-seq from various experimental platforms, as well as microarrays. We will also summarise the transcriptomic responses of not only the model but also useful crop and medicinal species, which can be used for the development of future stress-resistant cultivars by genetic and metabolic engineering (Figure 1).

## Alterations in plant transcriptomes during abiotic stress

Stress conditions can be defined as internal and external cues that affect the efficiency of physiological, metabolic, and molecular plant processes, leading to a reduction in the efficiency of energy-to-biomass conversion. They can be divided into abiotic and biotic ones (Umar et al., 2021).

**FIGURE 2:**
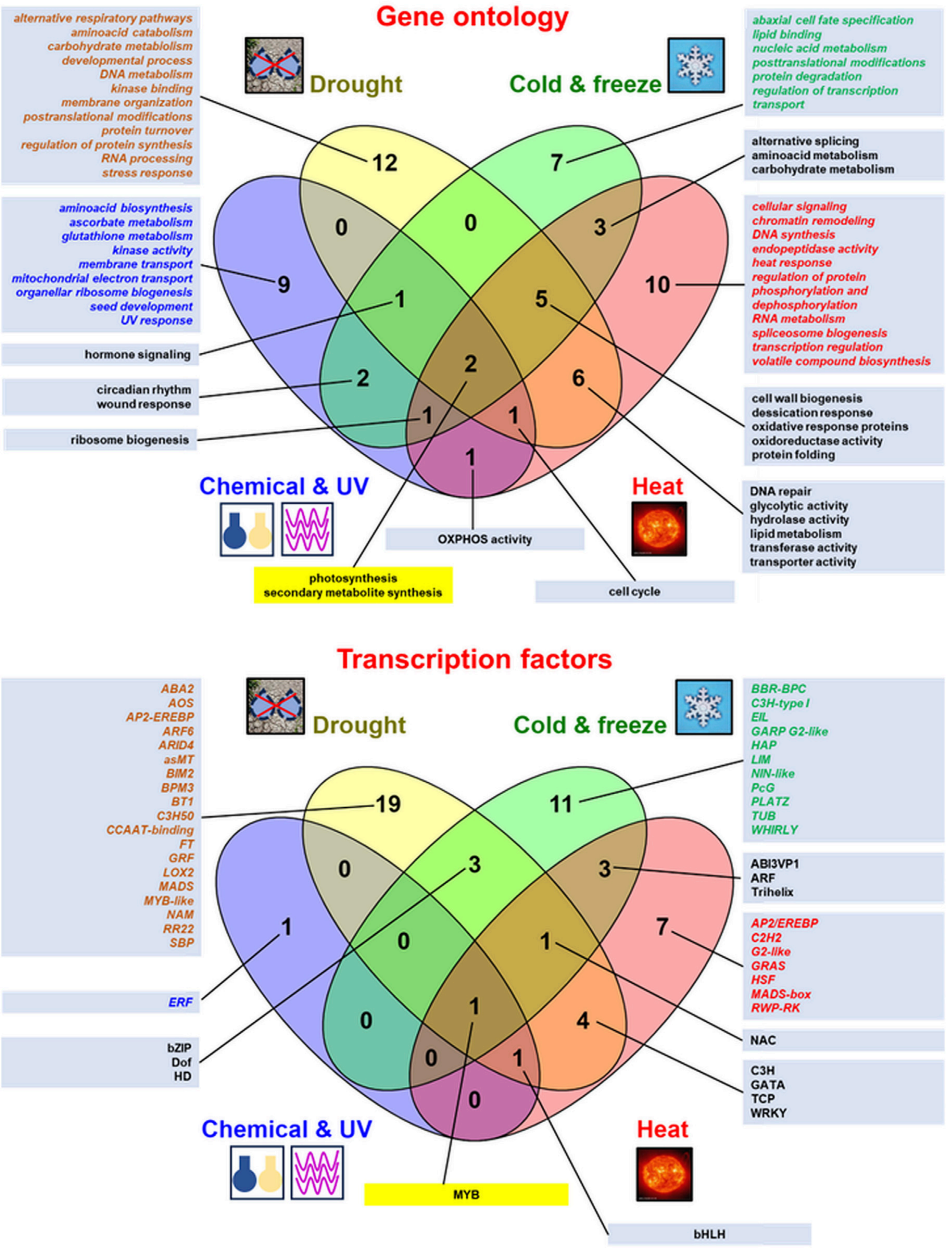
Comparison of the most relevant gene ontology (GO:) terms of plant genes and transcription factors active under abiotic stress conditions from various RNA-seq studies. The data were presented in Venn diagrams (drawn by Venny v. 2.1 from https://bioinfogp.cnb.csic.es/tools/venny/). The top enriched GO: terms (mostly relevant molecular functions and biological procesess) as well as transcription factors for regulated genes from the discussed studies were indicated. The data specific for the given stressor were denoted in *italics* and by different font colors (for the chemical treatment and UV radiation in blue, for drought in brown, for cold and freeze in green and for heat stress in red). GO: terms and transcription factors common for responses to all abiotic stressors were displayed within yellow text boxes. More details in the text.

Abiotic stress results from the action of multiple physical or chemical stimuli (Gull et al., 2019; Wang et al., 2020). A comparison of the plethora of enriched functional terms representing numerous genes and transcription factors (TFs) responsive to various abiotic stressors from RNA-seq studies is shown in Figure 2 and additional quantitative details on genes affected by abiotic stress from high-throughput transcriptomic studies are also given in Supplementary Table S1, where details on stress treatments and the respective references to the literature are also shown. The experimental studies discussed in this review showed the huge variability of the gene response under those conditions, even between similar treatments. However, the stress response involves not only functional terms/ TFs common for all abiotic stressors discussed here (representing differentially expressed genes [DEGs] for photosynthetic genes or genes controlling secondary metabolite biosynthesis as well as MYB TFs), but also specific ones for each treatment; they were also presented in Venn diagrams, although depending on the stressor (Figure 2). Leaves, which are involved in the metabolism of carbon skeletons and the capture of photosynthetic energy, belong to plant organs particularly affected by unfavorable environmental conditions. However, studies on the impact of stress on leaf tissues, contrary to roots, are still underrepresented (Berkowitz et al., 2021).

### UV radiation and chemical treatments

Variability of Arabidopsis leaf tissue transcriptomic responses under UV radiation, as well as under chemical treatments [e.g., antimycin A, 3-amino-1,2,4-triazole, methyl viologen, and salicylic acid (SA)] was characterized by Berkowitz et al. (2021). Responses of leaf tissues to these stressors were complex; for example, UV-affected genes were expressed mainly in the vasculature and epidermis. Treatment with 3-amino-1,2,4-triazole and SA downregulated genes for photosynthetic proteins in all tissues of Arabidopsis, while methylviologen upregulated the genes for the PS subunits, and UV radiation downregulated the photosynthetic genes in epidermis and upregulated them in mesophyll. Arabidopsis genes for oxidoreductase activity, porphyrin metabolism, plastid organization, and carbohydrate metabolism regulation were also affected by UV-B. Interestingly, Arabidopsis genes for proteins for chlorophyll biogenesis, protein folding, oxidoreductase and ligase genes, and glyceraldehyde-3-phosphate dehydrogenase were differentially regulated between UV-A and UV-B treatments. Tissues studied also showed distinct mitochondrial responses to antimycin A, which affected the expression pattern of respiratory genes, general oxidoreductase activity genes, glutathione transferase, as well as genes related to Ser/Thr kinase activity and membrane transport (Berkowitz et al., 2021).

Among DEGs upregulated in *Vaccinium corymbosum,* genes involved in plant hormone signal transduction were significantly enriched after 1 h, followed by genes involved in phenylpropanoid biosynthesis after 3 h, and genes involved in the flavonoid anthocyanin pathway after 6 h of exposure to UV-B. These results suggest that phytohormone-related genes contribute to the primary response to UV-B radiation. However, the highest number of DEGs appeared among *V. corymbosum* plants exposed to UV-B treatment for 24 h. Genes involved in proanthocyanidin and flavanol biosynthesis (*PAL1, 4CL2, CHS, CHI3, VcFLS* and *VcUFGT*) were upregulated by UV, and their expression level lasted a long time after 24 h of treatment (Song et al., 2022). These DEGs resembled Arabidopsis genes affected by radiation (Berkowitz et al., 2021). Common genes for UV-B response in Arabidopsis and *Pachycladon cheesemanii* included genes for amino acid, vitamin, pigment, and secondary compound metabolism (Dong, 2024).

The impact of UV-B radiation on the transcriptome of *Glycyrrhiza uralensis*, a potent medicinal species, was investigated by Zhang et al. (2018). Participation of amino acid metabolism and enzymes in secondary metabolite pathways in the response to radiation at five different time points was suggested. Genes for various amino acid metabolic pathways were differentially enriched depending on UV-B duration, however, DEGs for enzymes of cysteine and methionine metabolism were mainly enriched in all treatments.

*Polygonum cuspidatum*, a medicinal plant species used in traditional Chinese medicine, exhibits numerous medicinal applications (Ke et al., 2023). The impact of UV-C radiation on the transcriptome of *Polygonum cuspidatum* leaves was studied by Liu et al. (2019); more DEGs (including more downregulated genes) were involved in shorter radiation response. Furthermore, this was accompanied by an increased concentration of resveratrol (the most important stilbene phytoalexin polyphenolic compounds) in the leaves of *P. cuspidatum.* Resveratrol used in the nutraceutical industry exhibits anticancer and anti-ageing properties. Under UV-C radiation, upregulated DEGs coded enzymes involved in proanthocyanidin and flavanol biosynthesis, however, chalcone synthase (*CHS*) gene was downregulated. Furthermore, MYB, bHLH, and ERF TFs appeared to be potential regulators of resveratrol biosynthesis genes. These results will help to find more practical applications of resveratrol synthesis (Liu et al., 2019). Upregulation of PAL, C4H, 4CL, and STS enzymes by means of genome editing may be positively associated with the resveratrol levels (Hasan and Bae, 2017).

Under high light treatment (with the enhanced UV radiation) of ginseng (*Panax ginseng*), another medicinal species, affected DEGs in the leaf transcriptome were mostly involved in various metabolic pathways and in the stress response. Stress-responsive functions were enriched among 33% of the upregulated DEGs, and the high light intensity and/or ROS response was associated with another 6% of the affected DEGs (Jung et al., 2020).

Lettuce (*Lactuca sativa*) grown under greenhouse conditions usually contains a lover level of ascorbic acid (ASC), an essential antioxidant nutrient for human health, compared to field-grown plants. To investigate the effect of radiation on ASC level in plants, lettuce plants were treated with various UV-B doses. Numerous DEGs were identified within lowly and highly radiated plants (Zhou et al., 2023). It was suggested that the expression of *MIOX* (for *myo*-inositol oxygenase, a key enzyme in the *myo*-inositol pathway)*, APX,* and *MDHAR* may contribute to the indirect increase in the level of ASC induced by UV-B radiation (Lorence et al., 2004).

In general, plant transcriptomic responses to UV and chemical compound treatments were specifically enriched in genes for aminoacid biosynthesis, ascorbate and glutathione metabolism, kinase activity, membrane transport, mitochondrial electron transport proteins, organellar ribosome biogenesis and seed development (Zhang et al., 2018; Liu et al., 2019; Berkowitz et al., 2021; Song et al., 2022; Zhou et al., 2023; Dong, 2024). Interestingly, as shown on the Venn diagram, ERF proteins belong to TFs active under chemical treatment and UV radiation (Figure 2).

### Water deficiency (drought)

As drought belongs to factors that affect the largest part of crop productivity, the analysis of multiple molecular responses by omics studies would allow characterising the mechanisms of drought in crops that result in the search for stress-resistant cultivars (Verma et al., 2013; Shanker et al., 2014).

Analysis of sweet potato (*Ipomoea batatas*) transcriptome in drought allowed identification of various upregulated genes for ABA, ethylene, and JA biosynthesis, indicating the relevance of hormonal signaling in a water deficit. Genes for ABI phosphatase and Ca^2+^-ATPase were severely altered, while genes for SA synthesis appeared not affected (Zhu et al., 2019). Also in chickpea (*Cicer arietinum*) numerous genes for AP2-EREBP, bHLH, bZIP, C3H, MYB, WRKY or MADS TFs that regulate signaling regulation, secondary metabolism, or transition to the generative phase were involved in drought acclimation (Kumar et al., 2019). The transcriptomic response of *Phoebe bournei*, a Chinese wood species, to drought also used DEGs for plant hormone signal transduction in addition to genes for redox homeostasis (*POD, SOD,* and *CAT*), phenylpropanoid, flavonoid and porphyrin biosynthesis, starch and sucrose metabolism, chlorophyll *a*/*b* binding proteins, and genes for numerous TFs from 25 families (Li et al., 2022).

Recent investigation of transcriptomes of two rice (*Oryza sativa*) cultivars that varied with stress resistance revealed that genes for hormone signaling (in line with Kumar et al., 2019; Zhu et al., 2019; Li et al., 2022 studies), LEA proteins, proteins related to redox homeostasis and NAC and ZIP TFs played crucial roles in developing drought tolerance (Tyagi et al., 2023). Additionally, in the transcriptome of *Medicago falcata* seedlings, DEGs for hormone signaling (ABA biosynthesis, JA biosynthesis), nucleic acid helicases, and diverse genes for RNA polymerases and DNA repair proteins were enriched. In contrast, gibberellin biogenesis genes were antagonistically expressed compared to ABA-related genes (except for the *GID1* gene). Numerous TFs were also affected (Miao et al., 2015).

In *Ceratostigma plantagineum*, a resurrection species, studied by Xu et al. (2021), affected DEGs encoded proteins also active in hormone signaling, and in photosynthesis, stress response, amino acid catabolism, sucrose and fatty acid biogenesis, RNA processing and regulation, energy metabolism (distinctive in mild drought), protein modification and transport, and membrane organisation. Those data indicate the flexibility of primary and secondary metabolism in water shortage and re-watering, using, among others, an alternative respiratory pathway, the C3-CAM switch, and the GABA shunt. During global reanalysis of the *Glycine max* transcriptome, DEGs for proteins for hormone signaling, cell division, cell cycle, cell wall organization, stress responses, signal transduction, and regulation of gene expression, were regulated by progressing drought (De Oliveira-Busatto et al., 2022).

Mild drought-affected Arabidopsis genes code proteins that participate in ABA signaling, ROS biogenesis, response to osmotic stress, and also in cell wall remodelling and cell growth, among which multiple genes were previously not associated with drought-responsive mechanisms. Hormone signaling genes for PYRABACTIN RESISTANCE/ ABA receptors, two ACC oxidases and four ethylene response factors were downregulated and protein phosphatases 2C, HAB proteins, some ABA-responsive element-binding factors as well as some their target genes were all upregulated. Cell wall-loosening expansins, pectin lyases were also upregulated (Clauw et al., 2015). These data, which allowed insight into the transcriptomic landscape of six Arabidopsis accessions in drought, were further re-analysed by Benny et al. (2019), who underlined the importance of hydrogen peroxide, water deprivation, salinity, osmotic stress, and ABA-responsive proteins among upregulated genes. Transcriptomic analysis of rapeseed (*Brassica napus*), another representative of Brassicaceae, revealed that upregulated DEGs were related to the response to water deprivation, ABA signaling, osmotic stress, and other abiotic stimuli and lipid metabolism, as well as cutin, suberin, and wax biogenesis, fatty acid degradation, and secondary compound metabolism (Fang et al., 2022).

The multitude of various TFs was associated with the response to drought of *Dendrobium sinense*, an endemic species (Zhang et al., 2021). DEGs coded proteins for carbohydrate derivative and nucleotide binding, ATPase and oxidoreductase activity, pectin metabolism, and multiple TFs. Interestingly, more DEGs participated in a mild drought response, where detrimental downregulation prevailed (Zhang et al. (2021). Furthermore; Xia et al. (2024) broadened the analysis of *Dendrobium* drought responses by three additional species; the highest count of DEGs appeared in *D. fimbriatum*. Multiple DEGs among various *Dendrobium* species were involved in carbon metabolism and anthocyanin biosynthesis. Noticeable differences in the expression level of the *PEPC* gene (for phosphoenolpyruvate carboxylase) were associated with CAM and improved drought tolerance.

*Artemisia annua*, a medicinal species, is a potent worldwide source of artemisinin, an antimalarial compound. Attempts have been made to significantly increase artemisinin yield, and stress tolerance engineering would be one of such strategies. Drought response of *A. annua* leaf transcriptome employed many DEGs, including those coding for Δ-1-pyrroline-5-carboxylate synthetase, aquaporins, glyceraldehyde-3-phosphate dehydrogenase, LEA proteins, HSPs, glyoxalase I, glutathione-S-transferase, PR proteins, Ca^2+^-dependent protein kinases, as well as proteins involved in ethylene and oxylipin biosynthesis as well as NAC and MYB-related TFs (Vashisth et al., 2018).

Transcriptomes of two wheat (*Triticum aestivum*) varieties with contrasting drought resistance were compared; in stress resistant cultivars, the drought response involved genes for the synthesis of secondary metabolites and important transcription coregulators and TFs (Kumar et al., 2018).

Growth regulator 5-aminolevulinic acid (ALA) has been used to alleviate drought in grapevine (*Vitis vinifera*), by increasing antioxidative responses (Yang et al., 2023). Chlorophyll metabolism and photosynthetic apparatus were primarily affected by ALA, which uses synergistic mechanisms to alleviate drought. In the presence of ALA, alterations in the expression pattern of DEGs for chlorophyll biogenesis and Rubisco-related genes played an important role that allowed ALA to maintain cell homeostasis under water scarcity.

Little was known about the combined action of drought and cold on the plant transcriptome. Sharma et al. (2018) provided a comparative study of the impact of both stressors on the Arabidopsis transcriptome by meta-analysis of publicly available transcriptomic data. Responsive DEGs encoded proteins related to photosynthesis, respiratory burst, hormone response, signal transduction, and water deprivation, as well as some stress-specific genes. Furthermore, at least 43 diverse TFs were expressed in both treatments. Coolen et al. (2016) analysed Arabidopsis plants under drought combined with biotic treatments. The water deficit alone increased expression level of DEGs coding for proteins responding to oxygen-containing compounds and cell wall biogenesis. As the drought progressed, the more pronounced were the alterations in gene profiles. Each of the stressors induced specific expression profiles over time. In sequential stress application, Arabidopsis displayed transcriptome profiles similar to those of the second treatment, regardless of the nature of the first stressor. Overall, this study highlights the importance of stress signatures in identifying key molecular responses that act between various response pathways.

In general, hormone signaling pathways belong to the common terms for UV treatment (as discussed above; Figure 2; Song et al., 2022) and drought (Figure 2; Miao et al., 2015; Sharma et al., 2018; Kumar et al., 2019; Zhu et al., 2019; Fang et al., 2022; Li et al., 2022). The transcriptomic response in drought specifically affects a multitude of genes involved in alternative respiratory pathways, aminoacid catabolism and carbohydrate metabolism, developmental processes, DNA metabolism, kinase binding, membrane organisation, postranslational modifications, protein turnover, regulation of protein synthesis, RNA processing, and stress response. It is also specifically regulated by a particularly broad variety of TFs and transcriptional coactivators of various families (Figure 2; Kumar et al., 2018; Kumar et al., 2019; Benny et al., 2019; Zhu et al., 2019; Xu et al., 2021; De Oliveira-Busatto et al., 2022; Yang et al., 2023; Xia et al., 2024).

### Elevated temperature (heat stress)

Elevated temperature affects cereal productivity, particularly male generative organ development and pollen maturation and viability (Young et al., 2004; Asseng et al., 2011; Wu et al., 2015). Heat stress leads to an increase in the level of reactive oxygen species (ROS) and a simultaneous decrease in ROS scavenger activity, leading to biomolecul damage and apoptosis (Bita and Gerats, 2013; Guan et al., 2013). *Chrysanthemum* leaf transcriptomes were analyzed in heat with or without melatonin (to alleviate the consequences of heat treatment; Xing et al., 2021). Heat alone resulted in massive downregulation of DEGs. In contrast, heat combined with melatonin increased expression level of several DEGs. Melatonin affected *HSF* and *HSP*, starch and sucrose metabolism, cell signaling, chlorophyll, flavonoid, carotenoid biosynthesis genes, and genes for various TFs.

Comparison of microspore transcriptomes under heat in two tomato (*Solanum lycopersicum*) cultivars with contrasting stress tolerance revealed among upregulated DEGs at least 11 *HSP* genes. Increased expression of the *HSP* and *APX* genes pinpoints the key role of antioxidant enzymes in the heat response (Frank et al., 2009). Valdés-López et al. (2016) studied dynamics of root hair transcriptome in soybean (*G. max*) subjected to heat at various time points. Responsive genes were classified into 10 functional modules regulated by a few TFs. In general, heat affected the expression pattern of DEGs for protein folding genes, but also for genes coding proteins for chromatin remodeling, and lipid and ATP synthesis, indicating for the importance of controlling water/nutrient intake by roots and the relevance of maintaining high ATP level under heat response.

Rice leaf transcriptome under thermal shift was studied by Rashid et al. (2020). Multiple genes were affected for the abiotic stress response and metabolite biosynthesis. Among the DEGs affected, only a few photosynthetic/ OXPHOS genes as well as some genes for glycolytic enzymes were present. Chen and Li (2017) investigated *Brachypodium distachyon* leaf transcriptome in heat. Affected DEGs coded proteins that participate in alternative RNA splicing, spliceosome, and PS biogenesis, indicating an increased extent of such events in response to high temperature in order to synthesize protein isoforms alleviating heat detrimental effects.

To describe thermotolerance and protective mechanisms against thermal stress in desert species, the *Rhizya stricta* transcriptome, the evergreen shrub, was analysed at elevated temperature. Upregulated genes coded HSPs, chaperones, UDP-glycosyltransferase, aquaporins and transparent protein *testa 12*, suggesting the distinctness of thermotolerance in leaves of *Rhizya stricta*, which is controlled primarily by improving protein folding and preventing protein degradation (Obaid et al., 2016). *HSP* genes and genes for flavonoid biosynthesis were upregulated in leaves of three tea cultivars (*Camellia sinensis*), important medicinal species, under heat (Huang et al., 2024). Studied cultivars differed in stress tolerance and exhibited mainly upregulated DEGs under heat acclimation, however, among the affected DEGs in all cultivars, the genes for photosynthetic activity were the most notable. In heat-tolerant cultivars at elevated temperatures, genes for proteins containing the chaperone domain, including universal stress proteins (USPs), small heat shock protein sHSP18.1, chaperonin-like protein 2 (CLP2), and the LEA5 protein were preferentially expressed. Additionally, the level of flavonols increased in heat-tolerant varieties, accompanied by increased expression of *FLS* genes. Therefore, in accordance with Obaid et al. (2016), Xing et al. (2021) and Frank et al. (2009) reports, the study by Huang et al. (2024) highlights the importance of chaperones and secondary metabolism in the heat response.

Heat stress often acts simultaneously with water deficit. Mikołajczak et al. (2023) focused on investigating the impact of heat stress, drought, and their joining effects on transcriptome of barley (*Hordeum vulgare*) flag leaves. In medium-sized leaves, short heat stress (similarly to drought) affected multiple genes, regardless of the duration of the stress. However, under longer heat and drought, more DEGs were affected in large leaves. Investigated stressors affected mainly the *LEA* and *HSP* genes. Overall, Mikołajczak et al. (2023) provided novel data on the molecular mechanisms of barley flag leaf that determine the response to drought and heat. Furthermore, according to Mahalingam et al. (2022), the number of DEGs increased in barley heads in the stress-tolerant genotype as heat progressed. Heat response involved genes for transporter proteins, and ABA response, and resulted in differential expression of *LEA* genes in stress-sensitive genotype. In contrast, genes for nonspecific lipid transfer proteins and carbonate dehydratase were enriched in a stress-tolerant cultivar. Heat with drought resulted in a notable increase in DEG number only in the stress-sensitive cultivar. In particular, at least 900 TFs controlled transcriptional reprogramming in two barley cultivars in all treatments.

Cellular signalling, including hormone signaling, is particularly important in multiple treatments, when heat is combined with other stressors. Martin et al. (2021) pointed out the broadening range of DEGs affected by double treatment (heat and drought) of *Lolium temulentum*, which encoded proteins for cell signaling, cell cycle, organellar biogenesis, binding, transport, oxidoreductase and antioxidative activit, as well as chaperones and multiple TFs. When heat stress acted together with an elevated level of CO_2_, detrimental effects were only partially alleviated by deregulation of primary and secondary metabolism genes in flag leaves of durum wheat (*Triticum durum*) flag leaves. Most affected DEGs coded proteins involved also in cellular signaling but also in stress response and nucleic acid metabolism. In particular, genes upregulated by CO_2_ were often downregulated by heat (they coded, among others, photosynthetic and OXPHOS proteins, proteins for hormone signaling, enzymes of lipid and amino acid metabolism and the glutathione-ascorbate cycle, nucleic acid metabolism, and transport proteins (Vicente et al., 2019). Suzuki et al. (2016) investigated the impact of heat and salinity on Arabidopsis transcriptome. DEGs regulated by heat and salt stress were enriched in genes coded proteins for ABA signaling, stress response, developmental processes, protein metabolism, and DNA-dependent transcription. However, in heat, DEGs for ABA-responsive proteins, glyoxylase 17 and catalase, among others, appeared to be responsive, indicating the importance of the antioxidative response.

Arabidopsis leaf transcriptome under various stress conditions (salinity, osmotic stress, and heat) was also investigated by Sewelam et al. (2020). Of all these treatments, elevated temperature appeared to have the most notable effect on transcriptomic profiles. DEGs affected by heat covered induction of eleven *HSP* genes, late embryogenesis abundant (LEA) proteins, receptor-like kinases (RLKs), glutathione S-transferases, genes for carbohydrate binding, UDP-galactosyltransferases, membrane transporters, and programmed cell death (PCD), and genes for WRKY TFs. However, heat treatment repressed several cell cycle genes, ribosomal protein genes, and genes involved in DNA synthesis and repair. In particular, osmotic stress and heat acted antagonistically, while double treatment largely reprogrammed the gene expression pattern. Heat in combination with salinity and osmotic stress also induced numerous mitochondrial genes, presumably as a compensatory response to excessive protein degradation (Rurek et al., 2018).

In general, multiple stress treatment (including heat) in plant transcriptomes results in different and distinct transcriptomic responses under single treatments. In particular, although drought often accompanies heat, those stressors result in the upregulation of different gene sets in various plant species (Mahalingam et al., 2022; Mikołajczak et al., 2023). The transcriptomic response to heat stress specifically engages DEGs for cellular signaling (particularly in multiple stress treatments and under melatonin supplementation; Suzuki et al., 2016; Vicente et al., 2019; Martin et al., 2021; Xing et al., 2021), and also genes for chromatin remodelling, DNA synthesis, endopeptidase activity, heat response, regulation of protein phosphorylation and dephosphorylation, RNA metabolism, spliceosome biogenesis, transcription regulation, volatile compound biosynthesis as well as AP2/EREBP, C2H2, G2-like, GRAS, HSF, MADS-box and RWP-RK TFs (Figure 2; Frank et al., 2009; Obaid et al., 2016; Suzuki et al., 2016; Valdés-López et al., 2016; Chen and Li, 2017; Rurek et al., 2018; Vicente et al., 2019; Rashid et al., 2020; Sewelam et al., 2020; Martin et al., 2021; Xing et al., 2021; Mahalingam et al., 2022; Mikołajczak et al., 2023; Huang et al., 2024).

### Low temperature (cold, freeze)

Similarly to heat, also cold treatment and freeze result in plant growth and development aberrations, as well as direct inhibition of metabolic reactions. Due to the limited osmosis, cell dehydration and oxidative stress occur simultaneously with other detrimental responses. Most plants can gain tolerance to ice formation by gradually being exposed to reduced (non-freezing) temperatures under cold acclimation (Chinnusamy et al., 2007). In general, genes for the early cold response encoded a wide variety of TFs that regulate other gene expression (Gull et al., 2019).

Signal transduction and hormone signaling appeared also to be important for the low-temperature response. Amur vine (*Vitis amurensis*) transcriptomic response under cold used DEGs that encode proteins for signal transduction, transcription regulation, and alternative splicing. At least 38 major families of TFs (with 326 genes for TFs) involved in the regulation of cold response were detected, including several previously uncharacterized TF families homologous to Arabidopsis proteins (e.g., HAP2, ABI3VP1, ARF, PLATZ, LIM, atypical HD and MYB factors, BBR-BPC, zinc Dof, C3H-type I, EIL, GARP G2-like and Trihelix). Genes for CBL-interacting protein kinases participating in signal transduction were upregulated (Xu et al., 2014). Du et al. (2017) analysed the cold response of *Agropyron mongolicum* ABA receptors and upregulated genes for bZIP and NAC TFs. Most DEGs participated in carbohydrate metabolism, hormonal and phosphatidylinositol signaling, as well as biogenesis of numerous secondary metabolites. Also, the cold response of *M. falcata* focused on phytohormone and nodulation signaling, revealing some similarities with the drought replies; however, *ABF1*, *GID1,* and *AUX* genes were downregulated. Interestingly, *GH3* auxin-responsive gene was extensively upregulated in cold (similarly to *DIMI1*, but contrary to *DIMI2* and *DIMI3*, all of which encode important nodulation factors). Furthermore, at least 16 genes for MYB and 12 genes for NAC TFs were induced by cold, indicating their participation in cold tolerance (Miao et al., 2015). The transcriptomic response to cold in two rape cultivars (*Brassica rapa*) cultivars, varying with stress tolerance, also involved DEGs coding proteins for plant hormone signal transduction (MAPK signaling pathway) and also for photosynthesis, phenylpropanoid biosynthesis, lipid binding, plant-type cell wall, positive regulation of circadian rhythm, abaxial cell fate specification and basal TFs (Ma L. et al., 2019).

Arabidopsis chilling response covered almost half of expressed genes (Calixto et al., 2018; Liu et al., 2022). Cold response also involved genes for plant hormone signaling, but also for glucosinolate biosynthesis, transporter proteins, long ncRNA, RNA splicing, and spliceosome biogenesis, as well as multiple TFs from 52 families. Cold downregulated photosynthetic genes and short cold-induced quickly responding DEGs coding proteins for chloroplast organisation, ribosome biogenesis, and rRNA processing, which may activate cold tolerance. On the contrary, long cold affected DEGs for the cell response to hypoxia, fatty acid and flavonoid biosynthesis, as well as redox homeostasis and diverse *HSP* genes (Liu et al., 2022). Massive alternative splicing events occurred in the first few hours of cold treatment, including mobilisation of cold-responsive TFs, splicing factors, and selected RNA-binding proteins (Calixto et al., 2018). Interestingly, in *P. cheesemanii*, contrary to Arabidopsis, the cold response employed genes for glucosinolate metabolism. For Arabidopsis and *P. cheesemanii*, genes for wound-like circadian clock, as well as for secondary metabolite biogenesis, responded under the early cold response (Dong et al., 2023).

Cheng et al. (2019) described the effects of *Me*TCP4 (a specific cassava [*Manihot esculenta*] TF) overexpression in Arabidopsis plants during cold stress. Affected genes were classified as stress-responsive under all tested conditions, to DNA binding and TF activity in control, and to oxidoreductase, peroxidase, and antioxidative activity under cold treatment. Analysis of transcriptomic response of leaves of *Lavandula angustifolia* to cold revealed DEGs coding for photosynthetic proteins among affected genes. The most important functions of DEGs were also associated with the decreased stomatal conductance, ROS scavenging, and the development of cold tolerance. In general, these findings may allow for further engineering of cold tolerance in *L. angustifolia* to improve its medicinal potential, as this species is a source of aromatic compounds in traditional Chinese medicine (Li L. et al., 2023). In the leaf transcriptome of another medicinal species, *A. annua*, cold induced a multitude of genes for kinases, peroxidases, ABA biosynthesis, LEA and LEA-like proteins, various desaturases, glyoxalase I family protein, proteins for oxylipin and polyamine biosynthesis, Δ-1-pyrroline-5-carboxylate synthetase, NAC and MYB TFs (Vashisth et al., 2018).

Important metabolic regulations occur in plant organelles under cold acclimation. Naydenov et al. (2010) investigated the mitochondrial and nuclear transcriptomes of germinated wheat (*T. aestivum*). The upregulated genes encoded mitochondrial proteins, including Mn superoxide dismutase (SOD) and alternative oxidase (AOX); however, the level of expression of nuclear genes essential for mitochondrial biogenesis was visibly reduced. This indicates a fine-tuning of gene expression between mitochondrial and nuclear transcriptomes, executed by anterograde and retrograde signaling, affected by stress conditions.

On the whole, the cold response of plant transcriptomes specifically employs DEGs involved in abaxial cell fate specification, lipid binding, nucleic acid metabolism, post-translational modifications, protein degradation, regulation of transcription, and biomolecule transport. Cold response also specifically mobilizes a variety of TFs, including BBR-BPC, C3H-type I, EIL, GARP G2-like, HAP, LIM, NIN-like, PcG, PLATZ, TUB, and WHIRLY proteins (Figure 2; Naydenov et al., 2010; Xu et al., 2014; Miao et al., 2015; Du et al., 2017; Calixto et al., 2018; Vashisth et al., 2018; Cheng et al., 2019; Gull et al., 2019; Ma L. et al., 2019; Liu et al., 2022; Dong et al., 2023; Li L. et al., 2023). Hormone signalling is also affected by the cold response in numerous studies, including members of *Fabaceae* and *Brassicaceae* (Miao et al., 2015; Du et al., 2017; Vashisth et al., 2018; Gull et al., 2019; Ma L. et al., 2019; Liu et al., 2022).

## Plant transcriptomic responses under biotic stress

Under global climate alterations, crop species are gradually exposed to increasing biotic stress. The findings discussed below may help develop plant cultivars that are highly resistant to fungal, bacterial and virus infections. To cope with biotic stress, plants have developed defensive responses precisely induced by pathogen attack (Verma et al., 2013). Plant cells contain plasma membrane receptors, which recognize pathogen-associated molecular patterns (PAMP). Subsequently, PAMP-triggered immunity (PTI) usually stops the infection before it spreads throughout the plant. Due to the constant combat between pathogens and their victims, pathogens can neutralize PTI by secreting special effector proteins into the cytosol. In response, plants developed the ability to detect microorganisms by effector-triggered immunity (ETI). Interactions between intracellular receptors designed to recognize effector molecules produced by pathogens and effectors trigger a complex network of cell responses to achieve infection resistance (McDowell and Dangl, 2000). To protect against pathogens, plants use an “oxidative outbreak” that initiates a hypersensitive response (HR) limiting the pathogen spread (Sato et al., 2010).

**FIGURE 3:**
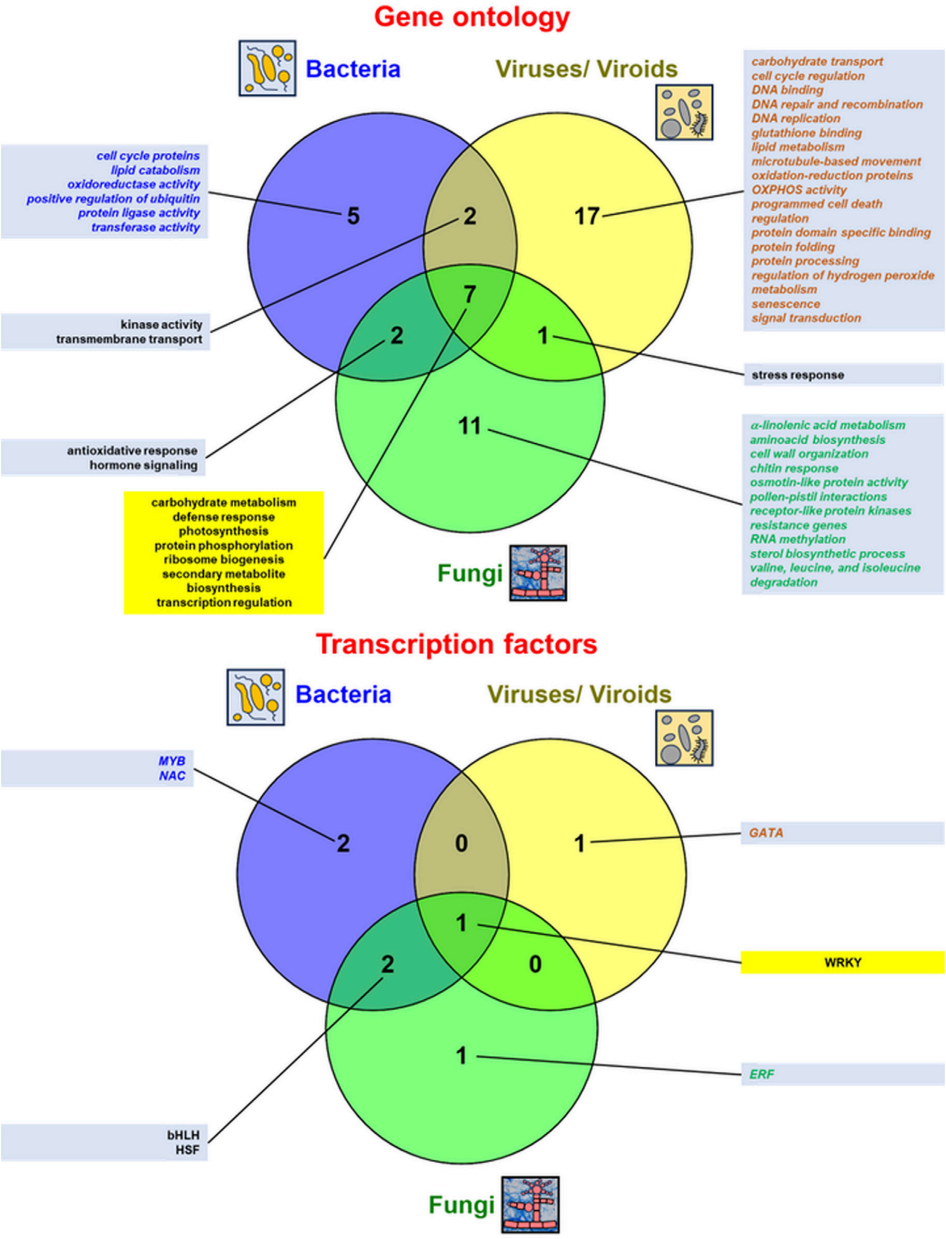
Comparison of the most relevant gene ontology (GO:) terms of plant genes and transcription factors active under biotic stress conditions from various RNA-seq studies. The data were presented in Venn diagrams (drawn by Venny v. 2.1 from https://bioinfogp.cnb.csic.es/tools/venny/). The top enriched GO: terms (mostly relevant molecular functions and biological procesess) as well as transcription factors for regulated genes from the discussed studies were indicated. The data specific for the given stressor were denoted in *italics* and by different font colors (for bacterial infections in blue, for virus/viroid infections in brown and for fungal infections in green). GO: terms and transcription factors common for responses to all abiotic stressors were displayed within yellow text boxes. More details in the text.

A comparison of the multitude of enriched functional terms representing numerous genes and transcription factors (TFs) that respond to various biotic stressors (fungal, bacterial, and virus/viroid infections) from RNA-seq studies is shown in Figure 3 and further quantitative details on genes affected by abiotic stress from high-throughput transcriptomic studies are also given in Supplementary Table S1, where details on various biotic treatments and references from the respective literature were given. Fungal, bacterial, and virus/ viroid infections involve common DEGs for carbohydrate metabolism, defence response, photosynthesis, protein phosphorylation, ribosome biogenesis, secondary metabolite biosynthesis, and transcription regulation. Biotic stress influence also expression patter of genes for hormone signaling/ signal transduction (more details below). All conditions of biotic stress mobilize WRKY factors responding to pathogen infection (Figure 3). Interestingly, terms for photosynthesis and secondary metabolite biosynthesis overlap with those for abiotic stress response; however, most functional terms and TFs in biotic infections differ from those of abiotic stressors (Figures 2, 3).

### Fungal infections

Pathogenic fungi can be divided into biotrophic, necrotrophic, and hemibiotrophic species (biotrophic in their early stages and necrotrophic in their later stages of the life cycle). Analysis of transcriptome of pumpkin leaves (*Cucurbita moschata*) infected with powdery mildew (*Blumeria graminis*) 24 and 48 h after the infection onset showed a downregulation of multiple DEGs, including genes coding for resistance to powdery mildew and various TFs for ethylene signaling. Numerous host photosynthetic genes were also regulated. Upregulation of photosynthetic genes after 48 h of infection was associated with the appearance of initial fungal hyphae, which was also shown in infected wheat (*T. aestivum*) (Guo et al., 2018).

The transcriptomes of wheat leaves infected with powdery mildew (*Erysiphales* species) and striped rust (*Puccinia striiformis*) were analysed by Zhang et al. (2014). In powdery mildew infection, DEGs coded proteins for α-linolenic acid metabolism, as well as phenylpropanoid, flavonoid, phenylalanine, tyrosine and tryptophan biosynthesis. However, striped rust upregulated DEGs for photosynthetic proteins and proteins for ubiquinone biosynthesis. These results indicate the participation of different genes in response to various fungi (Zhang et al., 2014). Coolen et al. (2016) investigated Arabidopsis transcriptome under infection of the necrotrophic fungus, *Botrytis cinerea*, alone or in combination with drought. DEGs responding to chitin, defence response, incompatible interactions, and RNA methylation were upregulated, and photosynthetic ones were deregulated up to 1 day after infection. Massive upregulation of core DEGs coding proteins that respond to oxygen-containing compounds according to the impact of drought. Kumar et al. (2022) compared two transcriptomic data sets of maize (*Zea mays*) silk infected with different fungal species. Set A contained data for silk samples affected by *F. graminearum* and *U. maydis*, while set B contained data from samples infected with *F. verticillioides* and *Trichoderma atroviride.* Interestingly, only 21 DEGs were found in all variants tested. Among these DEGs, peroxidase genes that control the lengthening of the germ tube to protect maize kernels from fungal disease were upregulated. The differential expression pattern was also found for the osmotine-like protein gene, which improves host defense and immune defense against stress, and for genes coding the receptor-like protein kinase subfamily. These genes appeared to be upregulated under infection with *Fusarium verticillioides*, *Fusarium graminearum*, and *Ustilago maydis* and downregulated when samples infected with *T. atroviride.*

Generally, host DEGs specifically regulated during fungal infections code proteins related to α-linolenic acid metabolism, aminoacid biosynthesis, cell wall organisation, chitin response, osmotin-like protein activity, pollen-pistil interactions, receptor-like protein kinases, resistance genes, RNA methylation, sterol biosynthesis process, as well as valine, leucine, and isoleucine degradation. SA signaling is believed to be mainly involved in resistance to biotrophic and hemibiotrophic pathogens. On the contrary, JA and ethylene signaling appeared to be indispensable for necrotrophic resistance (Pieterse et al., 2009; Guo et al., 2018). In addition, ERF belong to main TFs specifically regulating gene expression of plant host under fungal infections (Figure 3; Zhang et al., 2014; Coolen et al., 2016; Guo et al., 2018; Kumar et al., 2022).

### Bacterial infections

Analysis of rice (*Oryza indica*) infected with *Xanthomonas oryzae* revealed multiple upregulated genes that encode proteins involved in signal transduction, carbohydrate metabolism, and transcription regulation. On the contrary, the downregulated genes encoded TFs and proteins necessary for lipid catabolism, oxidative burst, and cell cycle (Kottapalli et al., 2007). Furthermore, analysis of the transcriptome of tomato (*Solanum lycopersicon*) infected with *Clavibacter michiganensis* allowed the identification of upregulated genes that encode proteins that also participated in hormonal signaling, but also in protein phosphorylation, and the plant defence response (including numerous TFs, such as WRKY, NAC, HSF, and CBP60). Resistance gene analogues (RGA) that included *RLK* genes were also upregulated. Exogenous treatment with SA resulted in induction of genes for WRKY TFs, therefore, SA-driven gene expression resulted in improved quality of the plant immune response (Yokotani et al., 2021).

Analysis of transcriptomes of rice (*O. sativa*) cultivars resistant and susceptible to infection with *Xanthomonas oryzaena*, which causes a cereal disease called bacterial leaf streak (BLS), revealed more DEGs among the infection-susceptible cultivar. Upregulations of genes encoding proteins involved in secondary metabolism, as well as participation of selected WRKY, NAC, MYB, and bHLH TFs in plant response to bacterial infection were notable (Lu et al., 2020). Deng et al. (2023) study on resistant (“*IBL2353*”) and susceptible (“*Ohio88119*”) tomato (*S. lycopersicum*) cultivars infected by *C. michiganensis* identified new gene families that participate in antibacterial defence. The key role of WRKY TFs in this process was revealed. At least 25 genes for proteins associated with the plant defense response and *WAKL20* gene (for the wall-associated receptor kinase similar to wall 20 and the only member of the WAKS subfamily that participates in innate resistance to pathogens) were upregulated in “*IBL2353*” cultivar. Viral-induced silencing of *WAKS20* gene in the “*IBL2353*” cultivar resulted in the appearance of susceptibility to *C. michiganensis* infection, suggesting an important role for the *WAKS20* gene in antibacterial defence (Deng et al., 2023).

In general, plant host genes specifically affected by bacterial infections code proteins related to the cell cycle, lipid catabolism, oxidoreductase activity, and positive regulation of ubiquitin protein ligase activity as well as transferase activity. Hormone signaling is also important for the antibacterial response of host plants (Kottapalli et al., 2007; Yokotani et al., 2021). Additionally, the host response to bacterial infections specifically mobilizes MYB and NAC TFs (Figure 3; Kottapalli et al., 2007; Lu et al., 2020; Yokotani et al., 2021; Deng et al., 2023).

### Viral and viroid infections

Plant transcriptomes also respond to the plethora of viral and viroids, which may leave their genome fragments within the host genome/transcriptome (Jo et al., 2018; Mifsud et al., 2022; Raza and Wu, 2022). The enriched functional terms for genes affected by viral and viroid infections are presented in Figure 3 and the summary of quantitative data from discussed experimental reports is available in Supplementary Table S1. Most viral proteins interact with host proteins, which promote the appearance of symptoms. Plants counteract this initial symptom development by using adaptive immunity; defence hormones are also active, resulting in hampering of virus biogenesis (Malavika et al., 2023).

Viroids are among the smallest and most infectious pathogens of crops, having short ssRNA genomes. They can affect host genes whose products participate in defense response, phytohormone signaling, cell wall modification, photosynthesis, secondary metabolism, transport, gene expression, and protein modification (Joubert et al., 2022).

Tobacco transcriptomes (*Nicotiana tabacum*) infected with seven genotypes of tobacco etch potyvirus (TEV) varying in fitness were compared. Host genes (including those for hormone signaling and RNA silencing-mediated pathways of plant defense) whose expression was correlated with the fitness level of TEV were examined (Cervera et al., 2018). The relevance of hormone signalling and signal transduction in the host response under virus attack was also pinpointed by other reports. Recently, Li Z. et al. (2023) focused on the analysis of antiviral response of bottle gourd (*Lagenaria siceraria*) under cucumber green mottle mosaic virus (CGMMV) infection. Affected DEGs were involved in the biosynthesis of secondary metabolites, hormone signal transduction (JA biogenesis), plant–pathogen interactions, and carbohydrate metabolism. Méndez-López et al. (2023) investigated the impact of the pandemic infection of the Pepino mosaic virus (PepMV) on two tomato cultivars. The *Sl*GSTU38 protein belongs to PepMV-specific susceptibility factors. The transcriptomes of healthy and virus-infected knocked out plants (*gstu38*) were examined. When *gstu38* plants were compared with healthy wild-type plants, some key stress-related genes (including those for WRKY TFs) were upregulated and genes for intracellular signal transduction proteins, various TFs, HSP70, and proteins involved in sugar metabolism and transport were downregulated. Among DEGs affected in both tomato cultivars, genes for peroxidases, various kinases, RNA binding proteins, resistance proteins, PDH, DNA repair, recombination proteins, and chaperonins and GATA TFs were also notable. Furthermore, the rice transcriptome was assayed during *planta* overexpression of the *Os*NF-YA protein displaying antiviral defense against rice stripe virus (RSV) and Southern rice blackstreaked dwarf virus (SRBSDV). Interestingly, the expression pattern of genes for JA biogenesis was decreased in plants overexpressing NF-YA under viral infection, due to interference between the NF-YA protein and TFs that regulate JA signaling (Tan et al., 2022). On the other hand, Arabidopsis cabbage leaf curl virus (CaLCuV) infection triggered the SA-dependent pathogen response and induced the expression of genes involved in PCD, genotoxic stress, DNA repair, and cell cycle (Ascencio-Ibáñez et al., 2008).Signal transduction is also crucial when plant viruses could alter host plant traits so that they modify their insect behavior. When *Myzus persicae* aphids were foraging in Arabidopsis and *Camelina sativa* plants, cauliflower mosaic virus (CaMV) infection appeared more severe than turnip yellows virus (TuYV) and affected DEGs that encode proteins for photosynthesis, oxidation reduction proteins, microtubule-based movement, as well as enzymes for JA, ethylene, and glucosinolate biogenesis. TuYV infection in Arabidopsis plants resulted in alterations in DEGs for carbohydrate transport, defence and stress response proteins (Chesnais et al., 2022).

Zhu et al. (2018) investigated the impact of cucumber mosaic virus (CMV) infection on hot pepper (*Capsicum annuum*) transcriptome. Affected genes were involved in stress and defence response, and plant-pathogen interactions. DEGs for chitinase, pathogenesis-related protein (PR), tobacco mosaic virus resistance protein (TMV), WRKY TFs, and jasmonate ZIM-domain protein, were upregulated after inoculation. Intron retention for *WRKY23* transcripts indicated a deep reprogramming in alternative splicing pattern under viral infection.

Zhou et al. (2019) studied transcriptomes of tomato (*S. lycopersicum*) plants grown from neutron-irradiated seeds and infected with tomato yellow leaf curl virus (TYLCV). Transcriptomes of plants grown from presoaked seeds were highly altered compared to those developed from dry seeds. Various doses of neutron irradiation affected the expression pattern of various DEGs. At least regulated DEGs that were common for all irradiated mutants encoded proteins for metabolism, transport, binding and responses to various stimuli, photosynthesis, and transcription. Spanò et al. (2020) applied the RNA sequence to study the transcriptomic profiles of tomato cultivars varying with resistance level, as well as their graft combinations, exposed to potato virus Y recombinant strain. Graft wounding and virus Y infection had various impacts on the tomato transcriptome, depending on genotype.

Roy et al. (2023) have used 3′RNA-seq to identify transcriptomic alterations in *N. benthamiana* plants infected by two strains of grapevine fanleaf virus (GFLV). During appearance of peak vein clearing symptom at 7 days post-inoculation (dpi), the genes involved in the immune response, gene regulation, and secondary metabolite production were over-represented. In other stages, DEGs related to chitinase activity, hypersensitive response, and transcriptional regulation were notable. Previously, Medzihradszky et al. (2019) investigated the transcriptomic reprogramming in the shoot apical meristem of *Nicotiana benthamiana* plants infected with Cymbidium ringspot virus (CymRSV). Upregulated genes coded proteins indispensable for cell defense, and downregulated DEGs were related to DNA replication and organization, shoot meristem development and plasmodesmata functions.

On the whole, plant host genes specifically affected under virus and viroid infections are related with carbohydrate transport, cell cycle regulation, DNA binding, DNA repair and recombination, DNA replication, glutathione binding, lipid metabolism, microtubule-based movement, oxidation-reduction proteins, OXPHOS activity, programmed cell death regulation, protein domain specific binding, protein folding and processing, regulation of hydrogen peroxide metabolism, senescence as well as signal transduction (with hormone signaling, highlighting relevance of JA signaling in host antiviral defence, e.g., Chesnais et al., 2022; Tan et al., 2022; Li Z. et al., 2023). Among TFs specifically mobilized under virus/viroid infection, GATA proteins were discernible (Figure 3; Ascencio-Ibáñez et al., 2008; Cervera et al., 2018; Zhu et al., 2018; Medzihradszky et al., 2019; Zhou et al., 2019; Spanò et al., 2020; Chesnais et al., 2022; Tan et al., 2022; Li Z. et al., 2023; Méndez-López et al., 2023; Roy et al., 2023).

## Discussion

Transcriptomic datasets, together with proteomic and metabolomic analyses, belong to the valuable and prospective components of modern systems biology in the functional network study (Figure 1; Cramer et al., 2011). In plant cells, in addition to the transcriptome being a product of nuclear genome expression, plastid and mitochondrial transcriptomes are also present (Barkan and Goldschmidt-Clermont, 2000; Daniell et al., 2016; Rurek, 2016; Rurek et al., 2018; Best et al., 2020a). They are dynamically shaped by multiple factors at multiple levels of biological diversity (Zhu, 2002; Wang et al., 2020). Studies on plant transcriptomes, which participate in stress response, may thus allow for better understanding how plants properly react to changing environmental conditions (Zhu, 2002).

### Plant stress studies employing RNA-seq

**FIGURE 4:**
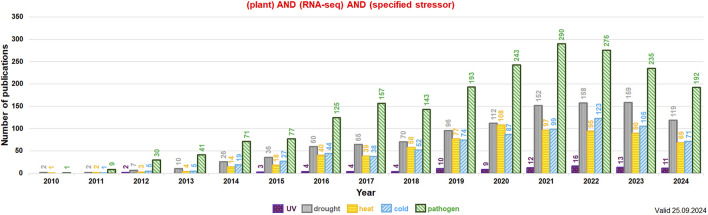
The detailed number of publications per year related to RNA-seq and specified stressors (*values indicated above each bar; subterms indicated by the various color and checking pattern in the histogram*). The key words used in the NCBI PubMed search (https://pubmed.ncbi.nlm.nih.gov/) included: plant, RNA-seq, and the given subterm (*indicated in the legend below the histogram*). The data for “UV” (*in dark magenta*), “drought” (*in gray*), “heat” (*in orange*), “cold” (*in blue*) and “pathogen” (*in green*; the joint data for bacterial, fungal and viral/viroid infections) were presented from 2010 onwards. The analysis was performed in September 2024.

Extensive research on the plant transcriptome is being developed at various levels. Transcriptomic data can be acquired both from differential expression studies in tissue/organs or even from single cells, and modern RNA-seq approaches. From 2010 the number of experimental reports on RNA-seq and stress response among diverse plant species increased almost exponentially. In total, between 2010 and 2024 at least 4,710 plant studies related to RNA-seq and UV, drought, cold, heat and pathogen response were published (Figure 4; the data valid to 25 September 2024). This indicates the growing interest of these topics in plant molecular biology. Interestingly, reports on pathogen attack and drought using RNA-seq dominate in number from at least 2014 and those for temperature treatments (cold, heat) are generally in the third place in each year indicated (Figure 4).

### Similarities and differences in transcriptomic response under various stressor conditions

The plant transcriptomic response depends greatly on the quality of the stressor and its duration, leading to metabolic flexibility (Supplementary Table S1; Wang et al., 2020; Xu et al., 2021; De Oliveira-Busatto et al., 2022). Commonly regulated genes under the action of almost all abiotic stressors often encode proteins for the photosynthetic apparatus and enzymes for secondary metabolism; interestingly, limited TFs (MYB mostly) overlap for all abiotic stressors (Figure 2).

In contrast, all conditions of biotic stress employ a large number of DEGs that represent a particularly broad landscape of enriched functional terms. DEGs for carbohydrate metabolism, defence response, protein modifications, ribosome biogenesis, transcription regulation, and secondary metabolism overlap both for bacterial, virus/viroid infections and for fungal infections across a plethora of host species (Figure 3). Furthermore, the WRKY proteins belong to universal TFs that regulate gene expression in biotic stress.

In general, photosynthesis and secondary metabolite biosynthesis belong to commonly enriched functional categories for all stress treatments discussed here.

From abiotic stressors, drought and heat stress employ the highest number of specific terms for these stressors (twelve and eleven terms, respectively, Figure 2). Drought stress appeared to be particularly detrimental at the transcriptomic level (Kumar et al., 2019; Zhu et al., 2019; Tyagi et al., 2023). Chemical treatments and UV as well as cold/freeze stress resulted in a lower quantity of terms specific to DEGs affected under action of those stressors. Furthermore, double stress treatments led to highly specific responses, sometimes increasing the number of DEGs (Zhou et al., 2019; Sewelam et al., 2020; Martin et al., 2021). It should be underlined that highly specific TFs from different families regulate gene expression under each abiotic stress conditions (with the highest number of TFs participating in drought and cold and freeze). Therefore, the most harmful stressors employ particularly numerous TFs within adaptive responses (Figure 2; Mahalingam et al., 2022).

Regarding biotic stress, virus and viral infections employ DEGs for host proteins with broadening functional categories that are specific only to those stressors. Less quantities of functional GO terms were also observed from the data representing fungal and bacterial infections (comparing with virus infections) were also notable. Genes for hormone signaling appeared to be important for both bacterial and fungal infections, and DEGs for signal transduction proteins were notably over-represented in virus/viroid infections (Figure 3). Overall, such a pattern differs from the one for abiotic stress treatments, where only a limited number of common GO terms for all treatments was evident (Figure 2). Furthermore, distinct TFs specifically regulate host gene expression under various biotic stressors, with MYB/NAC proteins mainly for bacterial infections and GATA and ERF factors for virus/virus and fungal infections, respectively (Figure 3).

As shown in Supplementary Table S1, stress-sensitive cultivars engage more DEGs in their stress responses; however, these patterns are highly tissue dependent (Mahalingam et al., 2022). Plant responses to biotic stress indicated the challenging importance of plant-microorganism interactions, which can generally influence stress tolerance (Rivero et al., 2022).

### Future outlines

From the data discussed within the present review, few conclusions for future actions can be presented. Hopefully, they will increase both the quantity and quality of the data. First, more organellar transcriptomes for important crop species should be analysed to better understand the responses of the plastome and mitogenome to stress and their relevance in plant developmental steps. Furthermore, due to the peculiar under-representation of transcriptomic data on stress in some plant organs, future studies should improve such biases (Best et al., 2020a; Best et al., 2020b; Berkowitz et al., 2021).

Most of the transcriptomic data discussed here came from plant material grown under controlled conditions. Therefore, field experiments that test the variations of multiple stress responses are still awaited. Additionally, both the complexity of plant viromes and impact of the plant microbiome on stress response at the transcriptional level should be widely investigated (Jo et al., 2018; Mifsud et al., 2022; Raza and Wu, 2022).

Due to the importance of DEGs for secondary metabolite synthesis in the analysed data, it would also be important (1) to investigate further the biogenesis of active compounds, (2) to better understand how metabolic pathways contribute to various stress acclimation strategies, and (3) to develop new more stress-resistant cultivars after genetic and metabolic engineering of medicinal and crop plant species. These actions should include modern methodological tools, for example, genetic engineering and gene editing, as well as metabolome engineering (Singh et al., 2016; Yan et al., 2020; Guo et al., 2021; Kumar et al., 2023; Dong, 2024).

## Glossary

ABA: abscisic acid
ABI3VP1: ABSCISIC ACID INSENSITIVE 3 VIVIPAROUS 1
ABF: ABSCISIC ACID RESPONSIVE ELEMENT-BINDING FACTOR
ACC: 1-aminocyclopropane-1-carboxylic oxidase
ALA: 5-aminolevulinic acid
AOS: allene oxide synthase
AOX: alternative oxidase
AP: APETALA
APX: ascorbate peroxidase
ARF: auxin response factor
ARID: AT-rich interactive domain
ASC: L-ascorbic acid
AUX: auxin receptor
BBR-BPC: Barley B Recombinant/Basic PentaCysteine
BIM: BES1-INTERACTING MYC-LIKE
bHLH: basic helix–loop–helix
BLS: bacterial leaf streak
BPM: BTB/POZ and MATH domain-containing protein
BT: BTB and TAZ domain protein
bHLH: basic helix-loop-helix
bZIP: basic (region) leucine zipper
CaLCuV: cabbage leaf curl virus
CAM: crassulacean acid metabolism
CaMV: cauliflower mosaic virus
CAT: catalase
CBP: calmodulin-binding protein
CDP: CAAT displacement protein (transcriptional repressor)
CGMMV: cucumber green mottle mosaic virus
C2H: zinc finger protein
C3H: zinc finger CCCH domain-containing protein
C4H: 4-cinnamate hydroxylase
CHI: chalcone isomerase
CHS: chalcone synthase
4CL: 4-coumarate: CoA ligase
Clp: caseinolytic protease
CLP: chaperonin-like protein
CMV: cucumber mosaic virus
CRISPR/Cas: clustered regularly interspaced short palindromic repeats/CRISPR-associated
CRK: cysteine-rich receptor-like kinase
CXIP: CAX-interacting protein
CymRSV: Cymbidium ringspot virus
DEGs: differentially-expressed genes
DIMI: DIMINUTO
Dof: DNA-binding with one finger
EIL: ethylene insensitive-like
ER: endoplasmic reticulum
EREBP: ethylene-responsive element binding protein
ERF: ethylene response factor
ETI: effector-triggered immunity
FLS: flavonol synthase
FRS: FAR1-RELATED SEQUENCE
FT: FLOWERING LOCUS T
G2-like: GOLDEN2-LIKE
GABA: γ-aminobutyric acid
GARP: GOLDEN2, ARR-B and Psr1 superfamily
GFLV: grapevine fanleaf virus
GH3: Gretchen Hagen3
GID: GA-INSENSITIVE DWARF
GRAS: GIBBERELLIC-ACID INSENSITIVE, REPRESSOR of GAI and SCARECROW
GRF: growth regulating factor
GSTU: glutathione S-transferase belonging to the *tau* class
HAB: HYPERSENSITIVE TO ABSCISIC ACID
HAP: CCAAT-box binding protein
HD: homeodomain
HLH: helix-loop-helix
HR: hypersensitive response
HSF: heat shock factor
HSP: heat shock protein
JA: jasmonic acid
LEA: late-embryogenesis abundant
LHC: light-harvesting complex
LIM: homeobox transcription factor
LOX: lipoxygenase
MADS: MINICHROMOSOME MAINTENANCE 1/AGAMOUS/DEFICIENS/SERUM RESPONSE FACTOR
MAPK: mitogen-associated protein kinase
MDHAR: monodehydroascorbate reductase
MIOX: *myo*-inositol oxygenase
MYB: myeloblastosis
NAC: no apical meristem/ATAF1/cup-shaped cotyledon
NF-Y: nuclear factor Y
NIN: nodule inception
OXPHOS: oxidative phosphorylation
PAL: phenylalanine ammonia-lyase
PAMP: pathogen-associated molecular pattern
PCD: programmed cell death
PcG: Polycomb group
PDH: proline dehydrogenase
PEPC: phosphoenolpyruvate carboxylase
PepMV: pepino mosaic virus
PLATZ: plant AT-rich sequence and zinc-binding protein
POD: peroxidase
PR: pathogen-related
PS: photosystem
PTI: PAMP-triggered immunity
RGA: resistance gene analogue
RLK: receptor-like kinase
ROS: reactive oxidative species
RR: response regulator
RSV: rice stripe virus
RWP-RK: transcription factor with RWPxRK motif
SA: salicylic acid
SBP: SQUAMOSA-PROMOTER BINDING PROTEIN
SOD: superoxide dismutase
SMRT: single-molecule real-time
SRBSDV: Southern rice blackstreaked dwarf virus
STS: stilbene synthase
TCP: bHLH DNA-binding domain
TEV: tobacco etch potyvirus
TF: transcription factor
TMV: tobacco mosaic virus
TUB: Tubby family of bipartite transcription factors
TuYV: turnip yellows virus
TYLCV: tomato yellow leaf curl virus
UFGT: UDP-glucose:flavonoid 3-O-glucosyltransferase
USP: universal stress protein
WAKL: wall-associated receptor kinase-like
WAKS: wall-associated kinases
WRKY: transcription factor family
WT: wild type
WUS: WUSCHEL
ZIM: zinc-finger inflorescence meristem
ZIP: leucine zipper
